# Copper Deficiency Myeloneuropathy Following Roux-en-Y Gastric Bypass in a 72-Year-Old Female

**DOI:** 10.7759/cureus.25109

**Published:** 2022-05-18

**Authors:** Zachary Kirkland, Ricardo J Villasmil, Jeffrey Alookaran, Mindy C Ward, David Stone

**Affiliations:** 1 Internal Medicine, Sarasota Memorial Hospital, Sarasota, USA; 2 Neurology, Sarasota Memorial Hospital, Sarasota, USA

**Keywords:** acquired peripheral neuropathy, nutrition education, roux-en y, gastric bypass surgery, copper myeloneuropathy

## Abstract

Following the implementation of gastric bypass for weight management, copper deficiency has become an increasingly recognized cause of myeloneuropathy. This condition typically presents with primarily sensory deficits leading to ataxia, similar to subacute combined degeneration from Vitamin B12 deficiency. We describe the case of a 72-year-old female patient who initially presented for insidious loss of sensation in her hands and feet, along with intermittent urinary retention. MRI findings included T2 hyperintensities of the dorsal cervicothoracic spinal cord. After identification of low serum copper, intravenous supplementation was started, with immediate improvement in symptoms by the time of discharge. Clinicians should recognize copper deficiency as a potential cause of progressive sensory neuropathy, particularly in patients with a history of gastric bypass.

## Introduction

Copper deficiency myelopathy (CDM) represents a relatively rare, often misdiagnosed acquired neurologic syndrome, the hallmark of which is dorsal column dysfunction and sensory ataxia [1]. CDM bears a striking similarity to subacute combined degeneration (SCD) from B12 deficiency: patients typically present with subacute gait difficulties with sensory ataxia and impaired fine motor movements of the hands, both related to subacute dorsal column degeneration. Long tract signs may also be present in addition to sensory loss. Patients often complain of draping or stocking neuropathy, raising concerns for syringomyelia during the initial evaluation. Copper is typically absorbed in both the stomach and the duodenum. With gastric bypass, particularly the radical restructuring of the gastrointestinal tract involved with Roux-en-Y bypass, patients are at increased risk of several nutritional deficiencies without proper guidance and adherence to dietary supplementation. As the number of these procedures increases, so too may the reported cases of copper deficiency and resultant CDM.

## Case presentation

A 72-year-old female with a past medical history of rheumatoid arthritis on methotrexate, hypothyroidism, and prior Roux-en-Y gastric bypass (21 years before presentation) presented for evaluation of bilateral upper and lower extremity numbness. Symptoms of bilateral hand sensory loss and paresthesias were first noted eight weeks before the presentation and were progressively worsening. Four weeks after the onset of her initial hand symptoms, she noted sensory loss in her feet, which progressed proximally to her knees. She denied weakness at first but reported trouble with fine motor skills, which she attributed to sensory loss and arthritis. However, on the exam, she had isolated mild bilateral hand weakness. Symmetrically diminished light touch and vibratory sensation of the hands and lower extremities were present. Position sense was largely intact but with some mild swaying with Romberg testing. Reflexes were normal to slightly reduced. She was without saddle paresthesia or gait disturbances but did report several episodes of urinary retention.

**Figure 1:**
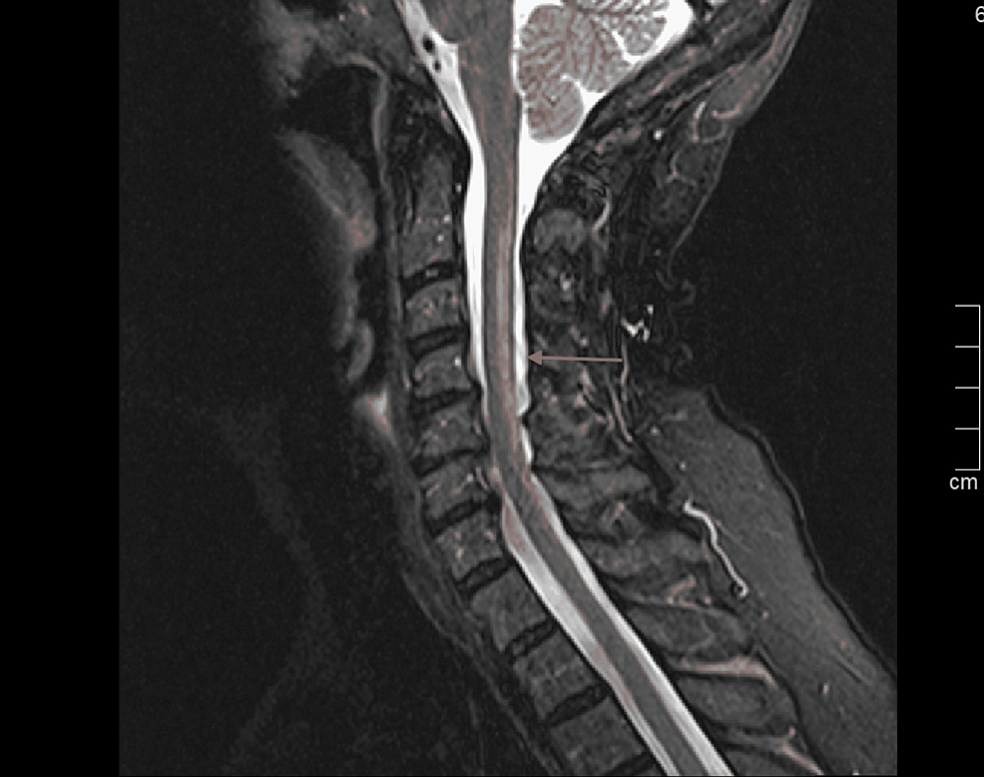
MRI cervical spine, Sagittal T2 view showing hyperintensity along with the dorsal columns at multiple levels (arrow). Some posterior disc bulges are also present.

**Figure 2:**
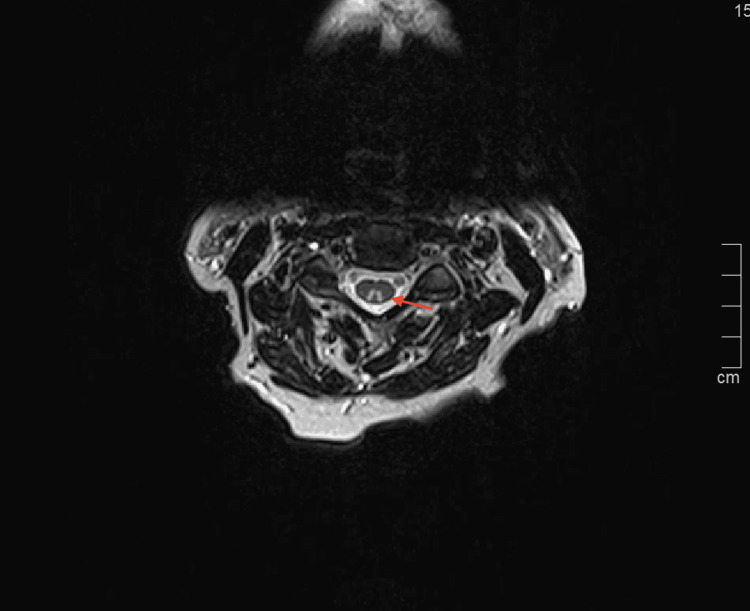
MRI cervical spine, axial T2 at the level of C3-4 showing dorsal column hyperintensity in a classic ‘inverted V’ pattern typical for subacute combined degeneration/copper myeloneuropathy (arrow).

She had previously seen her rheumatologist, who gave her a trial of gabapentin, which unfortunately did not provide relief. She also tried frequent chiropractic adjustments, also without significant relief. She was then referred for an outpatient MRI which showed extensive myelomalacia of the dorsal cervical spinal cord and was sent to the emergency department for further evaluation. Repeat MRI of the spine demonstrated bilateral symmetric T2 signal hyperintensity from C2 to at least T1 levels involving the posterior columns, a classic appearance seen in subacute combined degeneration (Figures 1, 2). She denied excessive zinc exposure/supplementation.

She had extensive laboratory evaluations, including normal cerebrospinal fluid (CSF) studies (with infectious and demyelinating studies), negative antinuclear antibody test (ANA), rapid plasma reagin (RPR), C-reactive protein (CRP), and erythrocyte sedimentation rate (ESR). She did not have laboratory evidence of hematologic abnormalities, renal disease, or liver dysfunction. Vitamin B12 level was slightly borderline at 245 pg/mL (reference: 193-986 pg/mL) and folate 45 ng/mL (3.1-17.5 ng/mL). Methylmalonic acid was elevated at 900 nm/L (reference: 87-318 nmol/L). Serum copper levels were extremely low at 10 mcg/dL (75-175 mcg/dL). Vitamin D and E levels were normal.

The patient revealed she stopped taking her bariatric vitamin supplements about a decade prior as she did not realize they needed to be continued. She was immediately treated with intravenous elemental copper infusions (4 mg for five days) as well as cyanocobalamin 1,000 mg intramuscularly daily (x 5 days then changed to monthly) and discharged on 2 mg oral copper gluconate daily and a daily bariatric multivitamin supplement. After several days of copper infusions, her symptoms began to improve, although she still reported persistent numbness in her fingertips and toes at discharge. At a follow-up appointment two weeks later, she noted continued improvement in her lower extremity sensory symptoms, a lack of worsening of her hand symptoms and a return to her baseline grip strength. Of note, she did have a reproducible Lhermitte’s sign in the office. At two months, she had some residual but stable sensory symptoms. Repeat copper level was 79 mcg/dL, B12 was 625 pg/dl, and her methylmalonic acid improved to 157 nmnol/L.

## Discussion

CDM most commonly occurs in the setting of prior bariatric surgery, particularly Roux-en-Y bypass [2], resulting in decreased gastrointestinal absorption in the stomach and duodenum. In this procedure, the stomach volume is drastically reduced, and the small intestine is reattached, leading to a marked alteration in the physiological uptake of nutrients. As this is a relatively new phenomenon, current epidemiological data is limited, although onset typically occurs at a mean age of 54 years with a 3:1 female predominance [3]. A history of Roux-en-Y bypass was present in over half of the reported cases [4]. Menkes’ Disease is an inherited copper deficiency due to fault ATP7A transporters in the duodenum. Copper is primarily used in two separate forms within the body: cuprous (1+) and cupric (2+), both of which are involved in a myriad of enzymatic redox reactions. While several proposed enzymatic deficiencies have been investigated, none have been definitively proven to cause CD [5]. Copper is carried throughout the body by ceruloplasmin, which rapidly degenerates in the absence of available copper, and thus low levels confirm a copper deficiency. While most copper is reabsorbed by the kidneys, approximately 0.5-1.5mg/day of copper is excreted in feces. However, the measurement of fecal copper is rarely useful as serum measurements are faster and more sensitive [6]. Other symptoms of copper deficiency include anemia, neutropenia, and myelodysplasia [7].

Typical imaging findings that should prompt testing of serum copper and ceruloplasmin levels include high T2 signal in the dorsal spinal cord, also found in subacute combined degeneration from B12 deficiency. While rare, combined copper deficiency and B12 deficiency may coexist. Confirmatory testing includes low urinary copper and low fecal copper. As bariatric surgeries are still a relatively new procedure, physicians must consider CDM in patients with sensory deficits and ataxia without other obvious causes. Generally, the prognosis is favorable, as most patients will respond well to parenteral administration of copper followed by oral supplementation with biweekly tapers, though no definitive guidelines exist for dosing. The Food and Drug Administration’s Recommended Dietary Allowance of oral copper for the average person is 0.9 mg/day2, although gastric bypass patients require higher amounts. It is imperative to advise patients to limit dietary zinc, as zinc will upregulate metallothionein, an intracellular chelator that readily and competitively binds copper [8]. There are no guidelines for the copper formulation, so clinicians should choose whichever formulation of copper is available at their facility (e.g., copper gluconate, chloride, etc.). Additionally, most patients will still exhibit mild sensory deficits despite aggressive treatments, so clinicians should advise patients their symptoms may not totally resolve. By recognizing CDM in its early phase, a further sensory loss may be mitigated.

## Conclusions

Following the advent of bariatric surgery, particularly Roux-en-Y bypass, vitamin and mineral deficiencies have become increasingly prevalent in those with normal diet. While the most common cause of mononeuropathy in these patients remains Vitamin B12 deficiency, our case demonstrates patients presenting with symptoms of subacute combined degeneration (i.e., dorsal column deficits) should also be evaluated for copper deficiency as these can co-exist. Emphasis should be placed on adequate supplementation in the immediate post-op setting and proper replacement of copper in those presenting with deficiencies. More data will be needed to establish preferred copper formulation, dosage, and length of therapy. This case demonstrates that copper deficiency can even occur decades after the procedure.
